# Creating a pathway for public hospital accreditation in Rwanda: progress, challenges and lessons learned

**DOI:** 10.1093/intqhc/mzz063

**Published:** 2019-07-19

**Authors:** Agnes Binagwaho, Kirstin Woody Scott, Theophile Dushime, Parfait Uwaliraye, Edward Kamuhangire, Dennis Akishuri, Denise Wanyana, Arielle Eagan, Laetitia Kakana, Joy Atwine

**Affiliations:** 1 University of Global Health Equity, Kigali, Rwanda; 2 Harvard Medical School, Boston, MA, USA; 3 Geisel School of Medicine, Dartmouth, Hanover, NH, USA; 4 Rwanda Ministry of Health, Kigali, Rwanda; 5 Management Sciences for Health, Rwanda Health System Strengthening Project, Kigali, Rwanda; 6 Vanderbilt University, Nashville, TN, USA

**Keywords:** Hospital accreditation, quality, performance-based financing, Rwanda

## Abstract

**Quality problem:**

Weaknesses in the quality of care delivered at hospitals translates into patient safety challenges and causes unnecessary harm. Low-and-middle-income countries disproportionately shoulder the burden of poor quality of hospital care.

**Initial assessment:**

In the early 2000s, Rwanda implemented a performance-based financing (PBF) system to improve quality and increase the quantity of care delivered at its public hospitals. PBF evaluations identified quality gaps that prompted a movement to pursue an accreditation process for public hospitals.

**Choice of solution:**

Since it was prohibitively costly to implement an accreditation program overseen by an external entity to all of Rwanda’s public hospitals, the Ministry of Health developed a set of standards for a national 3-Level accreditation program.

**Implementation:**

In 2012, Rwanda launched the first phase of the national accreditation system at five public hospitals. The program was then expected to expand across the remainder of the public hospitals throughout the country.

**Evaluation:**

Out of Rwanda’s 43 public hospitals, a total of 24 hospitals have achieved Level 1 status of the accreditation process and 4 have achieved Level 2 status of the accreditation process.

**Lessons learned:**

Linking the program to the country’s existing PBF program increased compliance and motivation for participation, especially for those who were unfamiliar with accreditation principles. Furthermore, identifying dedicated quality improvement officers at each hospital has been important for improving engagement in the program. Lastly, to improve upon this process, there are ongoing efforts to develop a non-governmental accreditation entity to oversee this process for Rwanda’s health system moving forward.

## Quality problem

Health systems worldwide have embraced hospital accreditation as a mechanism for bolstering patient safety and quality [1, 2]. Though interest in hospital accreditation in low-and-middle income countries (LMICs) is growing after decades of focus on high-income countries, uptake and success of accreditation initiatives in LMICs has been variable [3–6]. This paper summarizes the experience of Rwanda, an East African country of 12 million people, in developing its own national hospital accreditation system.

## Initial assessment

Though the 1994 genocide destroyed significant health infrastructure and capacity, Rwanda has demonstrated remarkable health gains since this tragedy [7]. In rebuilding, Rwanda’s Ministry of Health (MOH) aimed to develop and optimize access to quality care at its public hospitals in each district countrywide. This was accomplished by leveraging the infrastructure and evaluation methods employed by the country’s performance based financing (PBF) system [8], which started in the early 2000s [9]. After reviewing hospital quality gaps unveiled by PBF evaluations and learning of accreditation’s potential to address these gaps, the MOH determined that one of the country’s referral, tertiary hospitals (King Faisal) should take the nascent steps to achieve recognition by an international accrediting body. King Faisal was accredited by the Council for Health Service Accreditation of Southern Africa (COHSASA) in 2011 and has continued to pass subsequent re-accreditation evaluations by this external entity [10].

## Choice of solution

To meet identified quality standard gaps, Rwanda decided to broaden the COHSASA accreditation to all of its 43 district hospitals. Yet the cost of obtaining these additional site accreditations through an international body was prohibitively expensive. Thus, the MOH—in collaboration with international accreditation experts—developed a plan to implement its own national accreditation system.

## Implementation

The MOH requested a district-hospital situational analysis of ongoing quality improvement efforts to explore the impact of prospective accreditation expectations. Management Sciences for Health (MSH) technical experts guided this analysis, helping to build the ‘Essential Hospital Accreditation Standards’ framework. These standards were organized into five ‘focus areas’ aligned with national health sector priorities for assuring quality and safety (Table 1). Through its newly-formed Accreditation Steering Committee (ASC), the MOH tasked stakeholders to develop a three-tier system (Level 1–3) to meet accreditation progress and oversee the implementation of the national program.

**Table 1.** Rwanda’s hospital accreditation standards across five focus areas for level 1–3 status^a^

Focus area^b^	Critical standards^c^	Examples of additional standards^d^
*#1. Leadership Process and Accountability*	– Leadership for quality and patient safety – Compliance with laws and regulations	– Management of health information – Efficient admission and registration processes
*#2. Competence and Capable Workforce*	– Oversight of students and those in training – Training in resuscitative techniques – Credentialing of health professionals – Staff health and safety program	– Personal files available complete and up-to-date – Sufficient staff to meet patient needs – Staff performance management
*#3. Safe Environment for Staff and Patients*	– Management of hazardous materials – Coordination of infection prevention and control program – Barrier techniques are used (protective personal equipment) – Proper disposal of sharps and needles – Proper disposal of infectious medical waste	– Regular inspection of buildings – Stable safe water sources – Reduction of health care-associated infections (hand hygiene)
*#4. Clinical Care of Patients*	– Protocols for managing high-risk procedures and patients – Anesthesia and sedation used appropriately – Effective emergency triage – Essential emergencyequipment and supplies – Safe medication use	– Laboratory services available and reliable – Ambulance equipped – Patients educated to participate in their care
*#5. Improvement of Quality and Safety*	– Clinical outcomes monitored – Incident reporting system	– Patient satisfaction monitored – Staff satisfaction monitored

^a^The ASC endorsed a three-level status system for hospitals to demonstrate progress toward meeting the accreditation standards. A hospital with Level 1 status will have developed policies, procedures, and plans to address each of the critical standards and ensure that hospital staff have access to, and are aware of, such policies. A hospital with Level 2 status will have implemented these policies to promote effective risk-reduction activities. A hospital with Level 3 status will have captured data to demonstrate compliance with the standards and will have developed a monitoring system to track how the policies, procedures, and plans are improving quality of care.

^b^The five ‘focus areas’ are based on the Joint Commission International (JCI) Essentials of Health Care Quality and Safety Framework and hospitals are assessed for level of compliance to the pre-set standard in each category.

^c^Critical standards are defined as required by national laws and regulations or, if not met, may cause death or serious harm to patients, visitors, or staff.

^d^These are a subset of some of the additional standards across the five focus areas but are not deemed to be critical. For a complete list of the standards, please refer to the Rwanda Hospital Accreditation Standards, 2014. *Second Edition*.

## Evaluation

In 2012, the MOH identified five hospitals to participate in the accreditation program’s first phase (‘phase 1 sites’), aiming to collectively reach Level 1 status within 2 years. The sites were selected based on geographic diversity and their anticipated role expansion to become future provincial or referral sites.

In 2013, phase 1 sites underwent a 2-day baseline assessment. Showing aggregate scores across the focus areas ranging between 10.5%-26%, this assessment helped to elucidate barriers to achieving Level 1 status scores (minimum of 75% in each area, aggregate of 85%). The hospitals appointed dedicated accreditation facilitators, trained in quality improvement through MOH, to work on closing the notable gap between baseline and target scores.

By August 2014, four phase 1 sites achieved Level 1 status (overall scores between 85.8% and 92.6%); the fifth, only 1 point below the cutoff, later achieved Level 1. Building on phase 1, the MOH encouraged continued progress among the original sites and broadened the program to all remaining public hospitals. As of July 2018, 24 had achieved Level 1 and 4 had achieved Level 2.

## Lessons learned

### Barriers to accreditation

In an already resource-constrained environment, convincing hospital leadership and staff of the need for this additional program was challenging. Since accreditation was a new concept to most hospital personnel, the initiative was not initially prioritized. Turnover of leadership and staff who championed the work made it difficult to maintain momentum. Resource limitations, both financing and human resources, were also a challenge. Though initial funding limitations did not allow for a full-time accreditation facilitator, leaving tasks to be assumed by already-existing staff, ‘Quality Improvement Officer’ positions were eventually created at each hospital. External infrastructure constraints and resource variability– such as limited access to water or waste disposal – made it difficult for older hospitals to create adequate plans to meet accreditation standards. Fortunately, external limitations have continued to improve as Rwanda develops economically and strengthens its infrastructure.

### Enablers of accreditation

#### Pursuing a phased rollout

The ‘phase 1 site’ rollout allowed the MOH to refine its internal accreditation infrastructure—including surveyor training and the scoring system—to ensure standardization before expansion.

#### Giving flexibility to hospitals to write their own operational policies and procedures

While the ASC oversaw the list of accreditation-required essential standards, hospitals maintained flexibility in creating their own operational policies for achieving the standards. This fostered a sense of ownership among implementers, rather than asking them to implement a national policy not necessarily optimal for their context.

#### Linking accreditation to existing PBF incentives

Linking accreditation to the established PBF program legitimized accreditation and provided financial incentive for hospital leadership to pursue accreditation. Since cost influenced Rwanda’s decision to pursue its own program, merging with the existing PBF infrastructure maximized the limited financial and human resources available for hospital quality initiatives.

#### Building a cadre of accreditation champions

Providing competency-based training to facilitators was important, as few were initially knowledgeable about accreditation standards. On-site mentorship from MOH staff trained in quality improvement methodology initiated the growth of a cadre of experienced facilitators.

#### Future directions

As Rwanda’s internal governmental accreditation oversight structure, the ASC was developed to ensure a rapid, cost-feasible launch of the public hospital accreditation program. However, in order to reduce perceptions of potential conflicts of interest, oversight should ideally be provided by an external, non-governmental entity. Today, the MOH and partners are working to establish a non-governmental entity called the Rwanda Healthcare Accreditation Organization (RHAO). As Rwanda expands hospital accreditation standards to other public facility tiers and the private sector, the MOH will continue to support facilities advancing in their accreditation status and promote efforts to ensure that this translates into improved patient outcomes.

In summary, Rwanda’s experience demonstrates the feasibility of resource-limited countries developing national hospital accreditation programs. Rwanda will continue until all hospitals achieve accreditation, holding the promise of institutionalizing a health system-wide culture of quality improvement.
